# Ectopic Fetal Liver Tissue in the Placenta of a Twin Pregnancy: A Case Report and Review of Literature

**DOI:** 10.1155/2022/1966025

**Published:** 2022-06-13

**Authors:** Andrei Leucă, Pieter Demetter, Amélie Boulay, Katherina Vanden Houte, Valérie Segers, Laurine Verset

**Affiliations:** ^1^Department of Pathology, Centre Hospitalier Universitaire Brugmann, Place Arthur Van Gehuchten 4, 1020 Brussels, Belgium; ^2^Université Libre de Bruxelles, Avenue Franklin Roosevelt 50, 1050 Brussels, Belgium; ^3^Department of Pathology, Institut Jules Bordet, Rue Meylemeersch 90, 1070 Brussels, Belgium; ^4^Department of Obstetrics and Gynaecology, Centre Hospitalier Universitaire Brugmann, Place Arthur Van Gehuchten 4, 1020 Brussels, Belgium

## Abstract

Ectopic liver tissue represents a rare entity and is mostly attributed to events occurring during embryogenesis. Previous case reports documented the presence of fetal liver parenchyma within temporarily developed organs during pregnancy, such as the placenta or the umbilical cord. Moreover, the terminology of these benign findings varies from “ectopic liver” to “hepatocellular adenoma-like neoplasm” or “hepatocellular adenoma”. Ancillary tests performed on these lesions have shown positive immunohistochemical staining for hepatocellular origin marker HepPar-1. Only one recent case report comprising molecular analysis showed no beta-catenin gain-of-function mutation. We report a case of ectopic liver in one placenta of a twin pregnancy, with an updated review of literature.

## 1. Introduction

Human liver development begins at the end of the third week of the embryo development with the onset of the liver bud or hepatic diverticulum, appearing as a hollow midline outgrowth of endodermal tissue from the ventral wall of the future duodenum [1]. Failure during embryologic development could lead to liver tissue misplacement or choristoma, especially within the gallbladder, the hepatic ligaments, the diaphragm, but also in the thoracic cavity, the adrenal glands, the pancreas, the omentum, the spleen, and the esophagus [2]. Ectopic liver tissue has also been reported in the umbilical cord, as well as in the placenta [3–16]. However, these locations remain uncommon.

Here, we report the incidental finding of intraplacental fetal liver tissue from a diamniotic dichorionic twin pregnancy. We will show its histopathological and immunohistochemical characteristics and the potential pitfalls.

## 2. Case Presentation

A female patient in her thirties (G1 P0) was admitted in the Obstetrics Department for follow-up of a diamniotic dichorionic twin pregnancy, secondary to in vitro fertilization. On a routine blood test during the pregnancy, she was discovered a type 2 diabetes mellitus, for which she received insulin. Labor was induced at 31 weeks and 5 days of pregnancy because of premature rupture of membranes with suspected chorioamnionitis. Cesarean by low transverse section was performed and two viable male infants (1580 g and 2080 g, respectively) were delivered. The Apgar scores were 3 (5') and 6 (10') for the first newborn and 5 (5') and 8 (10') for the second newborn. In their postnatal period, the infants developed respiratory distress syndrome which was treated within the first three days, as well as jaundice, which responded successfully to phototherapy. One of the twins presented pyelic ectasia of the left kidney, without any other sign of malformation. One year after delivery, the infants and their mother were in good health.

Gross examination of the two formalin-fixed placentas was done using a standardized protocol, with sections at intervals no greater than 1 cm. The first placenta weighted 297 g and measured 17 × 17 × 1 cm, and the second one weighted 379 g and measured 19 × 14 × 1, 3 cm. The umbilical cords presented paracentral insertion on their respective discs, measured 19 cm and 16 cm in length, and contained three blood vessels each. Macroscopically, we noticed in the second placenta a subchorionic reddish nodule measuring 0,7 cm in its greatest dimension, at 1 cm from the insertion of the umbilical cord. No macroscopic abnormalities were observed in the first placenta.

**Figure 1:**
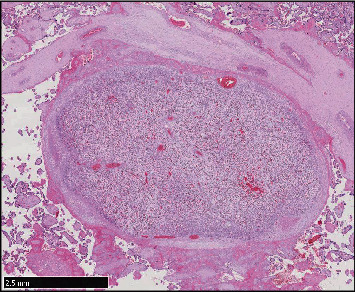
A well-circumscribed nodule within the placenta (hematoxylin and eosin).

**Figure 2:**
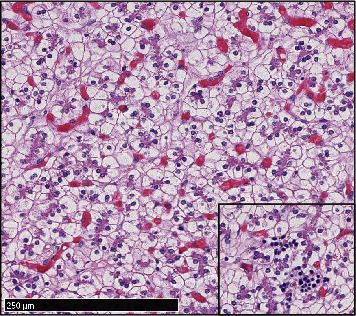
Trabecular-like structures of polygonal cells, with rare extramedullary hematopoiesis (hematoxylin and eosin).

**Figure 3:**
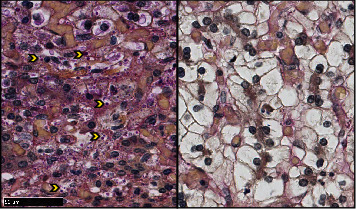
Glycogen deposits (arrowheads) were demonstrated using Periodic Acid-Schiff (PAS) and Periodic Acid-Schiff with diastase (PAS-D) stains.

Routine samples of the membranes, the umbilical cord, and full-thickness sections of the placental disk from both placentas were submitted. The microscopic examination of the nodule revealed a well-circumscribed lesion (Figure 1), surrounded by a fibrous capsule and composed of polygonal cells, with clear eosinophilic cytoplasm and small, round to ovoid nuclei. These cells formed trabecular-like structures, surrounded by a rich vascular network. Rare foci of extramedullary hematopoiesis were observed (Figure 2). No cytological atypia or mitoses were seen. No portal tracts were found. Some cells presented eosinophilic intracytoplasmic material, consistent with glycogen deposits, demonstrated by the Periodic Acid-Schiff (PAS) and Periodic Acid-Schiff with diastase (PAS-D) stains (Figure 3). Other microscopic findings were chorioamnionitis in both placentas and additional funisitis in the first placenta.

**Figure 4:**
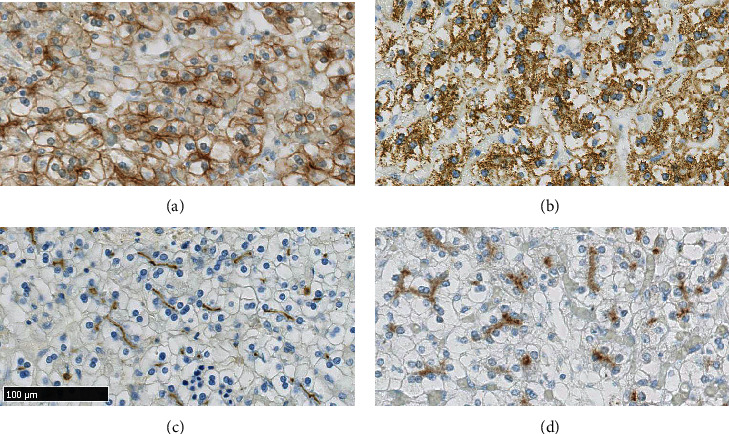
The polygonal cells express diffusely large spectrum keratin AE1/AE3 (a) and the hepatic differentiation marker HepPar-1 (b) antibodies. Polyclonal carcinoembryonic antigen showed expression in the canalicular pole of the hepatocytes (c). Glypican-3 antibody is expressed focally (d). (Dimension in figure 4(c) corresponds to all figures).

**Figure 5:**
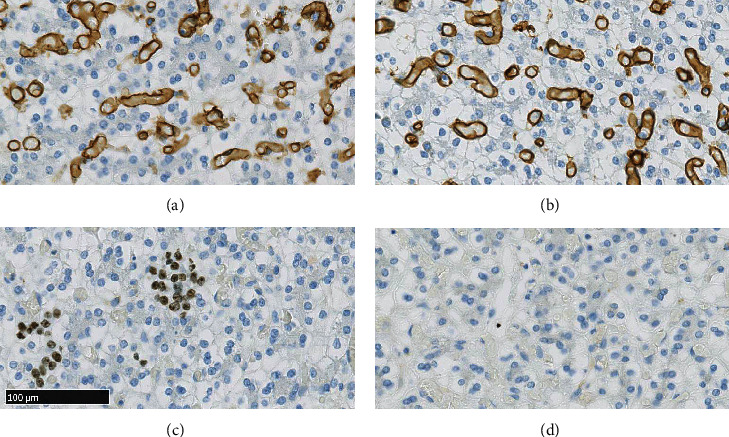
CD31 and CD34 markers showed diffuse expression in the sinusoid-like blood vessels (a and b, respectively). Ki67/MIB1 shows an increased proliferation index strictly limited to the extramedullary hematopoiesis foci (c). Cytokeratin 7 antibody expression is absent in the hepatocytes and proves lack of portal spaces (d). (Dimension in figure 5(c) corresponds to all figures).

Ancillary immunohistochemical studies were performed on BenchMark ULTRA system (Roche, Diegem, Belgium). Four micrometer sections were done on the FFPE tissue block. UltraView Universal DAB Detection Kit was used for all antibodies. Keratin AE1/AE3 (Ready-to-Use, Dako, Glostrup, Denmark) and hepatocyte paraffin-1 (HepPar-1, Roche, Diegem, Belgium) were diffusely expressed in the main cells (Figures 4(a) and 4(b)). Cluster of differentiation 10 (CD10, Ready-to-Use, Agilent, Machelen, Belgium) and polyclonal carcinoembryonic antigen (pCEA, 1 : 100, VWR/Klinipath, Leuven, Belgium) (Figure 4(c)) showed focal, canalicular-like pattern of expression. Glypican-3 (Clone 1G12, Sanbio, Uden, Netherlands) was also expressed focally (Figure 4(d)). Cluster of differentiation 31 (CD31, Ready-to-Use, Agilent, Machelen, Belgium) and cluster of differentiation 34 (CD34, Ready-to-Use, Roche, Diegem, Belgium) were positive in the blood vessels (Figures 5(a) and 5(b)). Ki67/MIB1 (1 : 200, Agilent, Machelen, Belgium) highlighted proliferative activity in the extramedullary hematopoiesis foci exclusively (Figure 5(c)). The cytokeratin 7 (CK 7, Ready-to-Use, Roche, Diegem, Belgium) antibody showed no expression in the lesion (Figure 5(d)).

## 3. Discussion

Although rare, ectopic liver tissue has been described in various organs (stomach, adrenal glands, pancreas, gallbladder, spleen, and lung) [7]. Moreover, this occurrence has been shown within temporary organs, such as the umbilical cord [3–6], with only 14 cases reporting an involvement of the placenta [7–16]. Heterotopic adrenocortical tissue in the placenta has also been reported [17–19], with a recent study challenging this origin by demonstrating hepatic differentiation [20]. A broad differential diagnosis was previously discussed in the literature [7–16], but for the current lesion we distinguished between primary placental nontrophoblastic neoplasms (chorangiomas) or metastatic disease (maternal hepatocellular carcinoma). These entities were excluded using the clinical data (no medical history of oncologic disease), the morphological aspect of the lesion (well delineated, clear and abundant cytoplasm, no cytological atypia, and no mitotic activity), and the immunohistochemical profile of these cells (expression of the large spectrum keratin marker AE1/AE3, hepatocyte differentiation marker HepPar-1, and the lack of proliferative activity, respectively).

One of the first case reports suggested that the lesion could represent a monodermal teratoma [8], but these entities are considered extremely rare and occur mainly in the sacrococcygeal sites [9]. However, in other studies, these lesions are considered hepatocellular adenomas [9–12] or ectopic liver [13, 14], with one paper using the terms “hepatocellular adenoma-like” and “benign hepatocellular tumor” [7]. We consider that our case represents an ectopic liver parenchyma. Indeed, as exposed previously, this benign entity may constitute a heterotopia of an embryonal liver tissue which has migrated in the placenta and whose development stopped [7, 10]. Strong immunohistochemical expression of CD 31 (as described in our case) was presented as an argument in favor of a true heterotopia [10]. The CD 34 expression in the endothelial cells has been previously described [12, 16] and demonstrated within developing liver parenchyma in the first 5 to 9 weeks of pregnancy [21]. Like in our case, glypican-3 protein expression by the hepatocytes has been described and is in favor of a fetal residue [12, 13, 22, 23]. Moreover, Saluja et al. suggested that in these early stages of development the liver parenchyma does not contain portal tracts, bile ducts, or classical lobular architecture [13]. Since we did not encounter these features in our case, we can hypothesize that the “implantation” of the ectopic liver in the second twin placenta occurred at the beginning of the pregnancy. However, in our case, the gestational age at the placental delivery was 31 weeks, compared to 16 weeks, for the previous mentioned study [13]. The most recent study observed a predominance of premature labor and delivery in a high majority of these lesions [16], which applies also to our case. We consider that this discovery could be related to the more frequent examination of the premature placentas compared to the ones delivered at term [24]. The rare reporting of these lesions can be explained by their size: in the literature it varies from 0,3 cm to 7 cm, with the majority of the lesions (like in our case) measuring less than 1 cm, which may explain the microscopic incidental findings in some cases. Nonetheless, the real incidence of ectopic liver within the placenta is still unknown. DeNapoli described ectopic liver tissue measuring 1,5 cm within a chorangioma [11]. Like in our case, some other authors described the presence of extramedullary hematopoiesis in the liver parenchyma [10–12]. Furthermore, as in the current study, there were two previous reports of a diamniotic dichorionic twin pregnancy [11, 12], but no clear association with this clinical aspect can be suggested. Yee et al. investigated the malignant potential of a placental hepatocellular adenoma, which showed no aberrant beta-catenin or glutamine synthase marker expression on immunohistochemistry, as well as a wild-type *CTNNB1* gene on molecular analysis [12]. In all the previous studies, as well as ours, it was proposed that these lesions be considered as benign. However, given the rarity of this type of lesions, further studies are necessary in order to establish their exact development, as well as their neoplastic potential.

## Data Availability

The histological and immunohistochemistry data used to support the findings of the study are included within the article.
